# Improving glycemic control in patients with type 2 diabetes mellitus through a peer support instant messaging service intervention (DiabPeerS): study protocol for a randomized controlled trial

**DOI:** 10.1186/s13063-022-06202-2

**Published:** 2022-04-14

**Authors:** Elisabeth Höld, Johanna Grüblbauer, Martin Wiesholzer, Daniela Wewerka-Kreimel, Stefan Stieger, Werner Kuschei, Philip Kisser, Elisabeth Gützer, Ursula Hemetek, Astrid Ebner-Zarl, Jürgen Pripfl

**Affiliations:** 1 Institute of Health Sciences, St. Pölten University of Applied Sciences, St. Pölten, Austria; 2 Institute of Creative\Media/Technologies, St. Pölten University of Applied Sciences, St. Pölten, Austria; 3 Department of Internal Medicine I, University Hospital St. Pölten, Karl Landsteiner University of Health Sciences, St. Pölten, Austria; 4 Bachelor Degree Program Dietetics, St. Pölten University of Applied Sciences, St. Pölten, Austria; 5 Department of Psychology and Psychodynamics, Karl Landsteiner University of Health Sciences, Krems an der Donau, Austria; 6 Fachbereich Versorgungsmanagement 3, Austrian Health Insurance Fund, St. Pölten, Austria; 7 Christian Doppler Forschungsgesellschaft, Vienna, Austria

**Keywords:** Type 2 diabetes mellitus, Instant messaging service, Peer support, Randomized controlled trial, Austria, Diabetes self-management (support), Social support, Glycemic control, Quality of life

## Abstract

**Background:**

Diabetes mellitus is one of the four priority non-communicable diseases worldwide. It can lead to serious long-term complications and produces significant costs. Due to the chronicle character of the disease, it requires continuous medical treatment and good therapy adherence of those suffering. Therefore, diabetes self-management education (DSME) (and support DSMES) plays a significant role to increase patient’s self-management capacity and improve diabetes therapy. Research indicates that these outcomes might be difficult to maintain. Consequently, effective strategies to preserve the positive effects of DSMES are needed. Preliminary results show that peer support, which means support from a person who has experiential knowledge of a specific behavior or stressor and similar characteristics as the target population, is associated with better outcomes in terms of HbA_1c_, cardiovascular disease risk factors or self-efficacy at a lower cost compared to standard therapy. Peer-supported instant messaging services (IMS) approaches have significant potential for diabetes management because support can be provided easily and prompt, is inexpensive, and needs less effort to attend compared to standard therapy. The major objective of the study is to analyze the impact of a peer-supported IMS intervention in addition to a standard diabetes therapy on the glycemic control of type 2 diabetic patients.

**Methods:**

A total of 205 participants with type 2 diabetes mellitus will be included and randomly assigned to an intervention or control group. Both groups will receive standard therapy, but the intervention group will participate in the peer-supported IMS intervention, additionally. The duration of the intervention will last for 7 months, followed by a follow-up of 7 months. Biochemical, behavioral, and psychosocial parameters will be measured before, in the middle, and after the intervention as well as after the follow-up.

**Discussion:**

Type 2 diabetes mellitus and other non-communicable diseases put healthcare systems worldwide to the test. Peer-supported IMS interventions in addition to standard therapy might be part of new and cost-effective approaches to support patients independent from time and place.

**Trial registration:**

ClinicalTrials.govNCT04797429. Registered on 15 March 2021.

## Administrative information

Note: the numbers in curly brackets in this protocol refer to SPIRIT checklist item numbers. The order of the items has been modified to group similar items (see http://www.equator-network.org/reporting-guidelines/spirit-2013-statement-defining-standard-protocol-items-for-clinical-trials/).

**Table:** 

Title {1}	Improving glycemic control in patients with type 2 diabetes mellitus through a peer support instant messaging service intervention (DiabPeerS) – study protocol for a randomized controlled trial
Trial registration {2a and 2b}.	ClinicalTrials.gov Identifier: NCT04797429
Protocol version {3}	21/11/2021, Version 6
Funding {4}	This investigator-initiated study is publicly funded by the Gesellschaft für Forschungsförderung Niederösterreich m.b.H, Austria (Life Science Call 2018, LS18-021)
Author details {5a}	^1^Institute of Health Sciences, St. Pölten University of Applied Sciences, St. Pölten, Austria ^2^Institute of Creative\Media/Technologies, St. Pölten University of Applied Sciences, St. Pölten, Austria ^3^Department of Internal Medicine I, University Hospital St. Pölten, Karl Landsteiner University of Health Sciences, St. Pölten, Austria ^4^Department of Psychology and Psychodynamics, Karl Landsteiner University of Health Sciences, Krems an der Donau, Austria^5^ Fachbereich Versorgungsmanagement 3, Austrian Health Insurance Fund, St. Pölten, Austria ^6^Christian Doppler Forschungsgesellschaft, Vienna, Austria
Name and contact information for the trial sponsor {5b}	Gesellschaft für Forschungsförderung Niederösterreich m.b.H, Hypogasse 1, 3100 St. Pölten, Austria; phone: +43 2742 275 70-0; email: office@gff-noe.at (formerly NÖ Forschungs- und Bildungsges.m.b.H)
Role of sponsor {5c}	The funding body (Gesellschaft für Forschungsförderung NÖ m.b.H.) is not involved in and has no influence on any study-related aspect of this study such as study design, management, analysis, interpretation, and dissemination of study results.

## Introduction

### Background and rationale {6a}

Diabetes mellitus is one of the four priority non-communicable diseases worldwide [1]. Globally, 463 million adults suffered from diabetes mellitus (9.3%) in 2019 and an increase of 51% of the prevalence is anticipated by 2045. Fifty-nine million adults are affected at the European level (8.9%) [2], while Austria shows a similar pattern: estimates range from 515,000 to 809,000 Austrians (7–11% of all inhabitants) suffering from diabetes [3]. In type 2 diabetes mellitus, which is the most common type of diabetes, hyperglycemia is the result of an inadequate production of insulin and insulin resistance, which means that the body cannot fully respond to insulin [2]. Type 2 diabetes mellitus is most commonly observed in adults over the age of 40 [4].

Diabetes can lead to serious long-term complications such as cardiovascular diseases, blindness, or the amputation of lower extremities as well as lower quality of life, poorer mental health like greater levels of depression, and reduced life expectancy [2]. Diabetes produces significant direct and indirect costs: 1.31 trillion USD in 2015 worldwide [5], 327 billion USD in 2017 in the USA [6], or 1.94 billion EUR even in Austria in 2014 [3].

Due to the chronic nature of diabetes, the disease requires continuous therapy, regular medical check-ups and good therapy adherence. Patient self-management behaviors in particular play an important role in the therapy of the disease: while healthcare professionals are responsible for only 5% of diabetes therapy, 95% are in the hands of the patients themselves, including, e.g., eating habits, physical activity, medication adherence, stress control, and disease monitoring [7]. Therefore, diabetes self-management education (DSME) (and support DSMES) play(s) a significant role in increasing a patient’s self-management capacity and improving diabetes therapy [8–11]. Self-management refers to the patient’s ability to manage symptoms, therapy, physical and psychosocial consequences, and lifestyle changes associated with living with a disease [12]. Consequently, DSME(S) is the process of facilitating skill, knowledge and abilities in diabetes self-care [11].

On the one hand, DSMES has been shown to improve clinical disease parameters like glycated hemoglobin (HbA_1c_) [13–15], which reflects the average plasma glucose level over the previous 8–12 weeks and is the preferred test for diabetes control [16], and even mortality [17]. On the other hand, research indicates that these outcomes might be difficult to maintain [18, 19] and seem to decline 1 to 3 months after the end of DSME(S) [20]. Consequently, effective strategies to preserve the effects of DSME(S) are needed.

Social support, defined as “an exchange of resources between at least two persons aimed at increasing the wellbeing of the receiver” [21], is a well-known predictor of diabetes self-management, improves self-efficacy, adherence and glycemic control [22, 23], and reduces healthcare costs [24]. Social support can be provided by several stakeholders like healthcare professionals, family members, friends or others living with the same disease (e.g., peers) [22].

Preliminary results show that peer support, which means support from a person who has experiential knowledge of a specific behavior or stressor and similar characteristics as the target population [25], is associated with better outcomes in terms of HbA_1c_, cardiovascular disease risk factors and self-efficacy [26–28] at lower cost [26] compared to standard therapy. Although those results are promising, research on peer support in diabetes care is still in its infancy [29, 30].

Peer support can be provided in various ways: face to face, telephone-based, web-based [29], or via other new technologies. New technologies in particular provide ideal and innovative means for peer support in diabetes management. Mobile Health (mHealth), which belongs to the group of information and communication technologies for health [31], supports medical practice using mobile devices such as mobile phones, monitoring devices, personal digital assistants, and other wireless devices [31]. Indeed, mobile phones have significant potential to serve as universal devices for diabetes management [32]. However, methodologically sound evaluation studies assessing the impact of mHealth are scarce [33, 34] and data security and other ethical concerns are often not sufficiently considered [32, 33]. Furthermore, most of the research on mHealth for diabetes focuses on support via apps or social media mainly provided by health professionals [34–37]. When it comes to support from peers or support provided via instant messenger services (IMS), less research is available. This is surprising because peer support IMS has particular advantages compared to other mHealth solutions:

- In 2020, the majority (72.9%) of Austrians aged 45–64 who were mostly affected by the onset of type 2 diabetes mellitus used IMS, while only 27.5% used other social media tools [38].
- Off-the-shelf IMS technology is available which safeguards personal data protection, privacy and the associated ethical principles (e.g., *mattermost*: https://mattermost.com/).
- Compared to standard therapy, long-term support via IMS can be provided easily [26] and promptly [37], is inexpensive [26], and requires less effort to attend [39].

Generally, there is a need for high-quality randomized controlled trials (RCTs) on the impact of IMS telemedicine on health [40]. Moreover, personal influences like genetic factors have not been properly considered yet: most existing studies focus mainly on average therapy effects, even though personalized medical approaches show that the response to therapy varies significantly between diabetic individuals [41, 42]. In addition, theories from social sciences and psychology suggest that, depending on individual differences such as the regulatory mode, some individuals benefit more from mHealth interventions than others [29, 43, 44]. Furthermore, IMS users differ in terms of extraversion, neuroticism or conscientiousness [45], which might induce different health benefits related to personality traits.

The DiabPeerS study aims to implement a peer-supported IMS intervention for patients with type 2 diabetes mellitus and to analyze the effects of the intervention on type 2 diabetes mellitus-related outcomes-especially HbA_1c_. Furthermore, DiabPeerS aims to gain further insight into the associations of personal influences on the individual use, acceptance and effects of peer-supported IMS interventions in diabetes therapy.

### Objectives {7}

The major objective of the study is to analyze the impact of a peer-supported IMS in addition to an antidiabetic therapy according to current Austrian guidelines [4] (hereafter referred to as “standard therapy”) on the glycemic control of type 2 diabetic patients. We hypothesize that peer-supported IMS improves maintenance of diabetes self-management and, hence, leads to longer-lasting improved disease-specific outcome parameters (HbA_1c_) and higher quality of life. In particular, our main hypotheses are:

H1: A peer-supported IMS intervention reduces HbA_1c_ of patients with type 2 diabetes mellitus compared to a standard therapy.

H2: A peer-supported IMS intervention helps to maintain diabetes self-management behaviors in patients with type 2 diabetes mellitus compared to a standard therapy.

H3: A peer-supported IMS intervention improves the quality of life of patients with type 2 diabetes mellitus compared to a standard therapy.

H4: A peer-supported IMS intervention improves medication adherence in patients with type 2 diabetes mellitus compared to standard therapy.

Furthermore, we want to assess the correlation between the specific personality trait of extraversion and the benefits of peer-supported IMS.

H5: Extraversion correlates positively with the benefits of peer-supported IMS intervention as measured by the frequency of IMS usage, quality of life, and HbA_1c_ levels.

### Trial design {8}

The presented trial is a parallel-group, two-arm superiority RCT to evaluate the efficacy of a peer-supported IMS intervention for patients with type 2 diabetes mellitus in Lower Austria. Peer support via IMS in addition to standard therapy is compared with stand-alone standard therapy.

Moderators play a central role in peer-supported IMS intervention because they initiate and moderate the IMS exchange and discussion. They are patients with several years of disease and therapy experience because they need personal and practical experience in order to provide supportive and credible coaching. All moderators receive prior training and guiding material (see {11a}). The moderators are supported by a dietitian at regular intervals and by other study team members if needed. One moderator is responsible for one IMS group consisting of 14 participants maximum.

The participants will be fully informed about all aspects of the trial and asked to sign a consent form. The participants will be randomized at a ratio of 1:1.

The duration of the intervention is 7 months, followed by a 7-month follow-up period. The intervention will be performed twice to expand the recruitment phase. During the follow-up, the intervention group is expected to use the IMS tool without guidance by health professionals and both groups receive standard therapy.

## Methods: participants, interventions, and outcomes

### Study setting {9}

The trial will be implemented in Lower Austria, which is a federal state in northeastern Austria. It is the country’s largest federal state and surrounds the Austrian capital Vienna. Lower Austria had 1,684,287 residents in 2020 [46], which reflects approximately 19% of all inhabitants of Austria [47].

Lower Austria has the second-highest prevalence of diabetes among all Austrian federal states. In this region, 5.7% of all residents above the age of 14 are affected by diabetes [3].

The trainings and medical examinations will be carried out at the St. Pölten University of Applied Sciences (located at the center of Lower Austria) and the hospitals in Wiener Neustadt (in the southeast of Lower Austria), Hollabrunn (in the northwest of Lower Austria) and Mauer/Amstetten (in the southwest of Lower Austria). This equal distribution throughout Lower Austria guarantees easy trial access for all participants. Biochemical analysis will be carried out at the University Hospital St. Pölten (located at the center of Lower Austria).

### Eligibility criteria {10}

Participants eligible for the trial must meet all of the following criteria during randomization:

#### Inclusion criteria participants (excluding moderators):

- Older than 40 years
- Living in Lower Austria
- Diagnosed with type 2 diabetes mellitus according to the Austrian definition [4]
- HbA_1c_ of ≥ 6.5% (48 mmol/mol) in the most recent measurement
- Undergoing standard therapy [4]
- Have been receiving oral hyperglycemic agents for a maximum of 3 years prior to the start of the trial (first prescription of a minimum of two packages per year to exclude wrong prescriptions)

#### Inclusion criteria moderators:

The inclusion criteria for moderators differ from the inclusion criteria for other participants because moderators are required to be more experienced in the management of their disease in order to be able to authentically share personal and practical experiences and to effectively support participants.

Therefore, they should (1) be interested, (2) be engaged in therapy participation and judged by the practice team as being generally adherent to therapy, (3) have the capacity and commitment to undergo the training, (4) have a complete understanding of the importance of data security and patients’ confidentiality, (5) cooperate with the dietitian and the study team if problems arise, (6) have the ability to fully understand the IMS strategy and the possibilities within the IMS tool, and (7) have several years of diseases experience (no newly diagnosed patients).

Therefore, their inclusion criteria are:

- Older than 60 years
- Living in the vicinity of the training location in St. Pölten, which means residing in St. Pölten, St. Pölten Land, Melk, Krems, or Lilienfeld
- Diagnosed with type 2 diabetes mellitus according to the Austrian definition [4]
- HbA_1c_ of ≥ 6.5% (48 mmol/mol) in the most recent measurement
- Undergoing antidiabetic therapy according to the current guidelines [4]
- Have been receiving oral hyperglycemic agents for a minimum of 3 years prior to the start of the trial (first prescription of a minimum of two packages per year to exclude wrong prescriptions)
- Engaged participation (regular participation) in the Austrian disease management program “Therapie aktiv – Diabetes im Griff,” which is provided by the Austrian Health Insurance Fund

#### Exclusion criteria for participants as well as moderators:

- Insulin dependency
- Hospitalization or vacation of more than 3 weeks during the intervention
- Eye disorders that severely limit vision and, hence, inability to read the display (e.g., proliferative retinopathy or macular edema)
- Severe illnesses such as kidney, liver, heart disease, malignant cancer, or neurological or mental illness which make a longer hospitalization likely
- Illicit drug use or non-medical use of prescription drugs determined by a single-question screening test for drug use and drug use disorders in primary care [48]
- Limited German language skills determined during the initial appointment concerning the informed consent
- Pregnancy

### Who will take informed consent? {26a}

Interested participants can register using an online registration form and a central study staff member - a dietitian or a nutritionist - will contact them afterwards.

During the initial appointment, which will be conducted online via MS Teams or face to face at the St. Pölten University of Applied Sciences, the staff member will explain the trial (background, protocol, different roles, importance of randomization and adherence) to potential participants and will review the consent form. Furthermore, the staff member will answer any questions arising. When the trial and its requirements are understood completely, the informed consent form will be signed by the participant. This can be done via electronic or handwritten signature.

All eligible participants must complete the consent procedure before enrolment and randomization. There are separate consent forms for general participants and for moderators.

#### Compensation

Participants and moderators will get a financial compensation in the amount of 50 EUR and a small gift after the last measurement. Furthermore, the results of the Bioelectrical Impedance Analysis measurements (BIA) in the context of body weight, body height, waist circumference, and blood pressure can be discussed with a dietitian for free after each measurement.

### Additional consent provisions for collection and use of participant data and biological specimens {26b}

No ancillary study will be conducted.

## Interventions

### Explanation for the choice of comparators {6b}

For the intervention group, we will use the instant messaging tool *mattermost* as android/iOS and web/desktop application, in addition to standard therapy. *mattermost* is an instant messaging service application that is installed on the cell phone and works similar to WhatsApp, i.e., people can use *mattermost* to send each other messages, photos, etc. Unlike WhatsApp, the messenger allows to track conversations and metadata of the participants, but *mattermost* is hosted in an ISO-certificated data center in Vienna for the duration of the project. Thus, it is hosted according to the European General Data Protection Regulation. The *mattermost* Starter Version (self-managed) is available for free, but the setup and secure hosting as well as the secure data extraction implementation is chargeable. For our participants, it has the look and feel of frequently used messenger tools, e.g., WhatsApp.

### Intervention description {11a}

The intervention is developed and conducted by an interdisciplinary team. Dietitians and moderators play an active role in the operational process of the intervention.

Dietitians are part of the team that develops the training curricula and is responsible for the training of the moderators and participants. They will supervise all moderators once a month via online meetings. During these meetings, the dietitians support moderators, e.g., if specific knowledge concerning diabetes care is needed or in case of technical problems.

Moderators act as facilitators and coordinators but they do not give medical or disease-related advice. In order to moderate communication and exchange in the IMS group effectively and authentically, personal and practical disease-related experiences of the moderator and the readiness to share these experiences with the group are essential. Moderators receive 9 h of training on the content to be shared via IMS and communication strategies to facilitate group exchange, peer support and behavioral change.

Participation in the clinical trial will last 15 months (1 month training/first measurement, 7 months intervention, 7 months follow-up). 196 participants will be randomly assigned to one of two groups: the intervention group or the control group. Participants who are randomly assigned to the intervention group will attend a 2 h technical training in connection with the first measurement. In the course of this training, they will learn how to use the *mattermost* app. After this training, they will join a *mattermost* group (instant messaging service group) with a maximum of 14 people for 14 months (7 months guided and seven without guidance). For the first 7 months of the intervention, an empowerment-based IMS communication strategy for diabetes self-management peer support has been developed.

Moderators are provided with a detailed handbook on web-based diabetes-related content and communication strategies to share this content with the group.

The IMS strategy combines two aspects that are essential for IMS diabetes self-management peer support: 1. a communication strategy that takes into account the special requirement of IMS communication, i.e., approaches to raise engagement and activities in the group, and 2. a diabetes self-management support strategy to make sure that all topics which are known to be of importance for diabetes self-management [49] are covered and shared in a way that enables and empowers participants in their disease management. Thus, the IMS communication strategy contains, on the one hand**,** the IMS strategy which also involves a communication strategy that considers the special requirements of IMS and peer-communication on how to react and act in certain situations, for example, to achieve engagement. On the other hand, it entails a didactic plan which outlines how, in what form and how frequently diabetic content shall be shared with the participants by moderators (e.g., web links, exchange of personal experiences/opinions). This IMS strategy will be operationalized in form of the training curriculum.

The moderator and the way she or he communicates with the group plays a vital role in guiding the group, setting the tone and creating an atmosphere of trust and providing support. By introducing the moderators to certain communication strategies that are also displayed in detail in the guidebook and can be reflected upon in the monthly meeting with the dietitian, a trusting atmosphere in the IMS group should be facilitated.

After the intervention, a 7 month follow-up will be conducted. During the follow-up, the intervention group may use the IMS tool in a self-organized manner, i.e., without guidance by the dietitian, and continue to receive their standard therapy.

Participants who are assigned to the control group will continue to receive their standard therapy but will not be assigned to a *mattermost* group.

The intervention will be conducted twice to increase the recruitment phase. The first intervention will start in April 2022. As soon as the first cohort enters the unaccompanied phase, a second moderated cohort will start - in both cases accompanied by an equally large control group. In total, we will be in the field for 23 months. With the start of the first intervention, we will have as many intervention and control teams with 14 people each as there are registrations. With the second intervention date, the remaining persons (196 in total) form further intervention and control teams.

### Criteria for discontinuing or modifying allocated interventions {11b}

Participants can revoke their willingness to participate at any time, even without giving reasons, and withdraw from the clinical study without this causing them any disadvantages for their further medical care.

The principal investigator may terminate a participation in the study prematurely without obtaining the participant’s consent beforehand. The reasons for this may be:

- Participants do not longer meet the inclusion requirements of the clinical trial
- The treating doctor reasons that continued participation in the clinical trial is not in the participant’s best interest
- The study coordinator makes the decision to terminate the entire clinical trial, or to terminate only one subject’s participation prematurely

The communication protocols (see also {12}) based on activities within the IMS tool *mattermost* are reviewed weekly. The results of these reviews are used-in addition to the planned analysis of the KPIs-to monitor the intervention group and to intervene if necessary. Thus, they help the study coordinator and selected study team members to stay informed and, in case of extraordinary events (e.g., mobbing), to decide whether to continue, modify, or prematurely terminate the work with certain participants, moderators, or communication concepts. If a participant does not adhere to the communication rules (netiquette) previously agreed upon in each IMS group, even after several discussions, he or she will be excluded.

### Strategies to improve adherence to interventions {11c}

The intervention consists of a peer-supported IMS intervention in addition to standard therapy. Therefore, adherence of participants to the IMS intervention pertains mainly to the IMS strategy, the measurements, and the initial training sessions.

The first step to improve adherence is the holistic explanation of the trial to interested participants during the initial appointment [50, 51]. In this appointment, sufficient time is scheduled in order to enable comprehensive understanding concerning the trial among participants.

Those who have greater responsibility - the moderators - will be individuals participating in the disease management program “Therapie aktiv – Diabetes im Griff” (https://www.therapie-aktiv.at), which is provided by the Austrian Health Insurance Fund. Hence, they are already proven to be generally adherent and engaged in their disease management. These moderators will positively influence their group members through the role model effect.

A dietitian experienced in counseling will support the moderators if problems arise and they will supervise all moderators once a month via online meetings. During the supervision, the dietitian will reflect on the past month and discuss the next steps of the IMS strategy with the moderators. If necessary, the communication expert can provide further assistance during these meetings.

Furthermore, we expect an additional positive social effect: participants will identify with their *mattermost* group and, in the next step, with the DiabPeerS trial. Hence, they will be interested in a successful intervention and participate actively. Also, frequent personal contact between the central study staff (EH, MH, AEZ) and the participants throughout the whole trial (initial appointment, four measurements, supervision meetings) will support adherence [50].

The IMS strategy (see {11a}), the core element of this trial, aims to improve diabetes self-management. Taking into consideration that regular medical appointments and monitoring are central in diabetes self-management, we assume that this increasing competence will support adherence to intervention as well.

Additionally, clinical visits will be designed as conveniently for the participants as possible [50, 51]. The participants will have easy access to the locations of training sessions and measurements: they can choose one out of four central locations in Lower Austria for their measurements. These locations are well known and easily accessible: the St. Pölten University of Applied Sciences and hospitals in Wr. Neustadt, Hollabrunn, and Mauer/Amstetten. These locations are equally distributed over Lower Austria from a geographical point of view and can be reached comfortably by car as well as public transport. The training sessions for the participants will take place after the first measurement at the same location. The training sessions for the moderators will be held at the St. Pölten University of Applied Sciences. Therefore, only individuals from St. Pölten or the surrounding areas are eligible to become moderators. If a participant misses one measurement, it is possible to make a new appointment within a certain timeframe.

Furthermore, study staff will call participants to schedule visits and send them reminders via SMS and e-mail 48 h before the appointment. During each measurement, participants and moderators will have the opportunity to discuss their results, such as body composition, with an experienced dietitian, which is not very common in Austria and will increase engagement [50].

Additionally, participants and moderators will receive a financial compensation of 50 EUR and a small gift after the last measurement.

### Relevant concomitant care permitted or prohibited during the trial {11d}

All participants should proceed with their standard therapy [4], and the moderators should continue to participate in the disease management program “Therapie aktiv – Diabetes im Griff” (https://www.therapie-aktiv.at) [52] during the trial.

It is also possible for participants to subscribe to the disease management program “Therapie aktiv – Diabetes im Griff” during the trial. This program was launched in Austria in 2007 and aims to organize long-term and high-quality care for patients with type 2 diabetes mellitus. It is voluntary and free of charge for physicians as well as for patients. It implements evidence-based clinical guidelines and supports patient empowerment, regular medical examinations, and lifestyle advice [52]. At the end of 2020, 14,976 patients with type 2 diabetes mellitus and 296 doctors in Lower Austria - or 93,099 patients with type 2 diabetes mellitus and 1917 doctors at the national level - participated in this disease management program according to internal data of the Austrian Health Insurance Fund.

### Provisions for post-trial care {30}

Trial participation entails only minimal risk compared to standard therapy alone, which will be continued after the trial. Therefore, no special provisions for post-trial care are included.

### Outcomes {12}

Data will be collected four times over a period of 14 months: at baseline (T0), 3 months after the start of the intervention (T1; T0+ 3 months), at the end of the intervention (T2; T0+ 7 months), and after the follow-up (T3; T0+ 14 months), including the following collection of biochemical, psychosocial, and behavioral parameters during all four data collection points.

The primary outcome is the level of HbA_1c_ [%] measured using TOSOH G8 (Sysmex Austria GmbH).

Secondary outcomes are:

Psychosocial parameters:

- Social support will be measured using the “Fragebogen zur Sozialen Unterstützung” (F-SozU) [53]: the F-SozU operationalizes social support as perceived or anticipated support from the social environment. The short form consists of the following subscales: “emotional support,” “practical support,” “social integration,” “stress from the social network.” The F-SozU involves of 14 items using a five-point Likert scale with the endpoints “1”(does not apply) and “5”(accurate).
- Self-efficacy will be measured using the “General Self-Efficacy Scale” (GSE) [54]: the GES consists of ten items designed on a four-point Likert scale with the endpoints “1” (not at all true) and “4” (exactly true) and assesses optimistic self-beliefs to cope with several challenges in life.
- Depression will be measured using the “Patient Health Questionnaire-9” (PHQ-9) [55]: the PHQ-9 asks for all nine criteria of depression as defined in the Diagnostic and Statistical Manual of Mental Disorders (DSM-IV) using a four-point Likert scale with the endpoints “0” (not at all) and “3”(nearly every day).
- Diabetes distress will be measured using the “Diabetes Distress Scale” (DDS) [56]: the DDS includes for dimensions of distress (“emotional burden,” “regimen distress,” “interpersonal distress,” “physician distress”). The DDS consists of 17 items using a six-point Likert scale with the endpoints “1” (not a problem) and “5” (a very serious problem).
- Quality of life will be measured using the “Short-Form-Health Survey” (SF-12) [57]: the SF-12 includes eight dimensions (“physical functioning,” “role limitations due to physical problems,” “bodily pain,” “vitality,” “general health perceptions,” “social functioning,” “role limitations due to emotional problems,” “mental health”). The summary scores “physical component summary” and “mental component summary” (0–100 scales) can be calculated from the specified scales.
- Diabetes knowledge will be measured using the “Diabetes Knowledge Test” (DKT) [58] with forward and retranslation: the DKT consists of 20 statements about diabetes which have to be rated as “true,” “false,” or “don’t know.” Based on the answers, a difficulty index (percent of patients who scored correctly) is calculated.

Behavioral parameters**:**

- Medication adherence will be measured using the “A14-scale” [59]: the A14 consists of 14 items of non-adherent behaviors phrased in a non-threatening and non-judgemental way using a five-item Likert scale with the endpoints “4” (never) to “0” (very often).
- Dietary behavior and alcohol consumption will be measured using a food frequency questionnaire (FFQ), which assess “dietary behavior” and “alcohol intake” during the past 4 weeks with 53 items and the endpoints “never” and “more than 4 times a day”.
- Physical activity will be measured using the “International Physical Activity Questionnaire Short Form” (IPAQ-SF) [60, 61]: the IPAQ-SF asks seven questions to assess “vigorous-intensity” and “moderate-intensity” physical activity as well as “walking” and “sitting.” Participants indicate the time in minutes or hours for each activity level. Based on this information, three levels of physical activity (low, moderate, high) are calculated and expressed in metabolic equivalent of task (MET) minutes per week.
- Diabetes self-management behaviors will be measured using the “Summary of Diabetes Self-Care Activities German” (SDSCA-G) [62]: the SDSCA-G focuses on the past 7 days related to the diabetes self-care activities “nutrition,” “physical activities,” “blood glucose testing,” “foot care,” and “smoking.” The SDSCA-G consists of 11 items and participants mark the number of days the mentioned behavior was performed on an eight-point Likert scale with the endpoints “0” (0 days) to “7” (7 days). While the first ten items are calculated to a score and four sub scores (diet, exercise, blood-glucose testing, foot care), the eleventh item focuses on smoking habits.
- Clinic and communication visits (health professional visits in the past 6 months, hospital stays in the past 6 months).

Biochemical parameters:

- Fasting blood glucose [mg/dl] as measured using Cobas 8000 (Roche Austria GmbH)
- Total cholesterol [mg/dl] as measured using Cobas 8000 (Roche Austria GmbH)
- High-density lipoprotein (HDL) [mg/dl] as measured using Cobas 8000 (Roche Austria GmbH)
- Low-density lipoprotein (LDL) [mg/dl] and triglycerides [mg/dl] as measured using Cobas 8000 (Roche Austria GmbH)
- Blood pressure [mmHg] as measured using Boso Medicus uno OA
- Body height [cm], body weight [kg] and body fat [%] as measured using Seca mBCA 555. The device measures body weight with a scale, body height with ultrasound length measurement and body composition by the voltage drop of the alternating current in one step.
- Waist circumference [cm] to calculate the waist-to-height ratio as measured using an ergonomic, step less, and extendible measuring tape

Additionally, demographic data and personality traits will be surveyed in the baseline assessment (T0). Demographic data include age, gender, education, living arrangement (marital status), income, employment status, immigration background [63], and clinical information (age at diagnosis, on insulin, other medical therapies, list of prescribed medications). Personal traits will be assessed using the Big Five Inventory German (BFI-2) [64]. The BFI-2 captures “extraversion,” “agreeableness,” “conscientiousness,” “negative emotionality,” and “openness,” each represented by three sub-facets. The BFI-2 consists of 60 items using a five-item Likert scale with the endpoints “1” (strongly disagree) to “5” (strongly agree).

Additionally, participation in the disease management program “Therapie aktiv – Diabetes im Griff” as well as pregnancy and illicit drug use / non-medical use of prescription drugs will be evaluated at each measurement.

Communication data (real-time):

As part of the data export solution of the IMS communication data, an export script is provided in which individual rooms - these correspond to the groups assigned for the experiment - are listed. Such communication protocols containing the communication data of the individual groups can be retrieved on a daily basis. These data include, for example, the persons who have sent a post, sent texts and files, timestamps, or quotes. This information will be used to quantify the IMS communication activities.

The posts will be evaluated by content analysis to explain and interpret the results of (H2) diabetes self-management behaviors, (H3) quality of life of participants, and (H4) medication adherence of participants. The codebook will be developed in collaboration with medical professionals for the coding of adherence and non-adherence behaviors. The quantified data will then be made available for the in-depth analysis of questionnaire data and medical scores.

According to past research [65–67], personality traits such as extraversion can influence our communication behavior: extraverted individuals speak more often, make more eye contact, smile, and nod more frequently than introverted persons. (H5) Therefore, we assume that extraversion will correlate positively with the number of posts as well as the number of reactions and emojis to posts from others.

### Participant timeline {13}

**Fig. 1:**
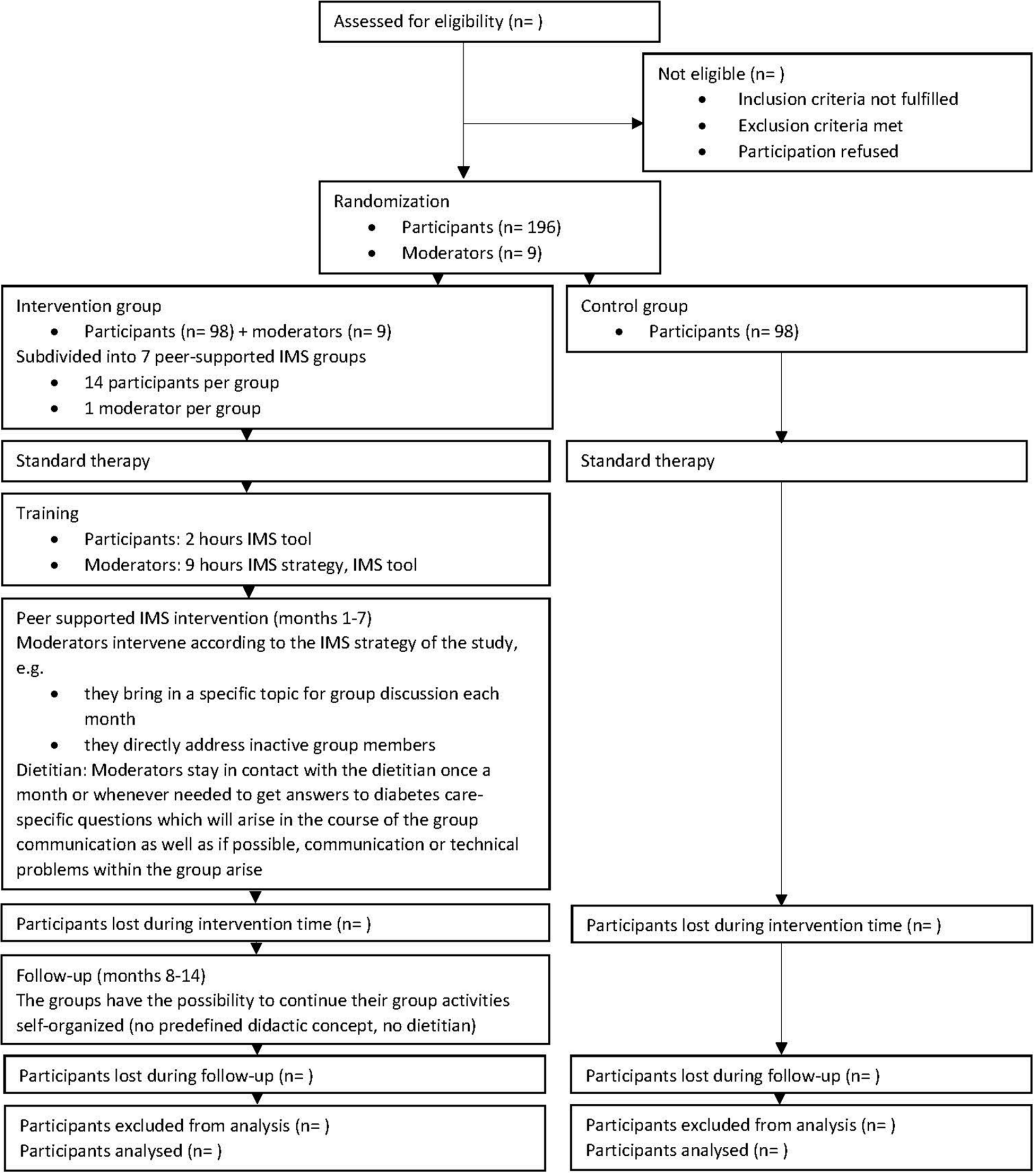
Flow diagram DiabPeerS

**Fig. 2:**
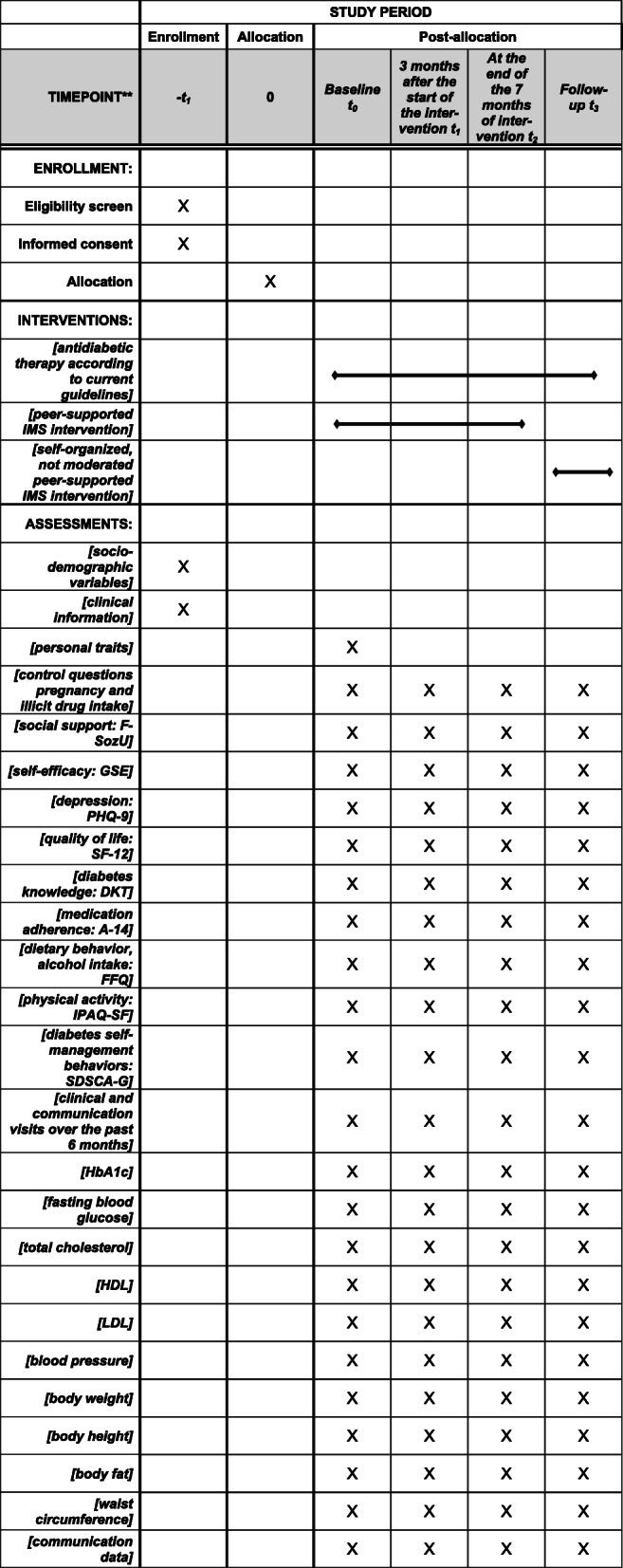
SPIRIT figure DiabPeerS

The flow diagram showing the participant timeline through the DiabPeerS study is presented in Fig. 1. The SPIRIT figure for this trial is given in Fig. 2.

### Sample size {14}

An a priori calculation was performed using G-Power [68] for the primary outcome variable HbA_1c_, using an ANCOVA at 5% level of significance and 80% power. Based on previous research, we assumed a small to medium effect size for group differences in HbA_1c_ means (partial eta squared = 0.05, which corresponds to an effect size *f* = 0.229) [69–71]. Considering a 30% dropout ratio, we will include 98 randomly assigned participants in each group (196 total). The intervention group will be subdivided into seven IMS groups made up of 14 participants and one moderator each. Therefore, nine moderators (seven plus two for the compensation of possible drop-outs) will be additionally recruited (205 persons in total, 196 participants plus nine moderators). Although the moderators will participate in the assessments, their data will not be included in the analyses.

### Recruitment {15}

The Austrian Health Insurance Fund will perform a FoKo database (Folgekosten database) query based on defined inclusion and exclusion criteria to identify potential participants in Lower Austria.

The FoKo database is a social insurance data warehouse that is used by all health insurance providers in Austria. FoKo combines several IT (accounting) systems of Austrian health providers in one instrument and thus enables various analyses. FoKo collects data related to medical assistance, medication, and incapacity to work as well as in-patient stays or patient transport and dental treatment. FoKo has registered approximately 1.2 million people eligible for services of the Austrian Health Insurance Fund in Lower Austria.

Based on FoKo database queries of the years 2018 and 2019, approximately 6000 potential participants will be identified. The data of the Mother-Child-Booklet, which is part of the Austrian pregnancy and childhood examinations, allows to identify pregnant women (with gestational diabetes) in order to exclude them from the study [72]. The Austrian Health Insurance Fund will contact all identified patients with type 2 diabetes mellitus by personal letter while strictly considering data privacy. Additionally, the Austrian Health Insurance Fund will inform all relevant physicians and internists in Lower Austria about the trial and ask them to inform their patients about the possibility to participate in the trial. Furthermore, the diabetes-specific self-help organizations ADA (https://aktive-diabetiker.at/) and ÖDV (https://www.diabetes.or.at/home/) will inform their members about the trial. Additionally, leaflets will be disseminated at the Customer Service Centers of the Austrian Health Insurance Fund and via the consortium member institutions’ online channels, e.g., their websites.

## Assignment of interventions: allocation

### Sequence generation {16a}

Eligible participants who have signed the informed consent will be randomized into the peer-supported IMS intervention group or the control group in a 1:1 ratio. Random assignment will be conducted using virtual urn randomization during the initial appointment.

### Concealment mechanism {16b}

For the allocation of the participants, a simple urn-based randomization is carried out. For this purpose, an urn, in our case a digital card set, is filled with 196 cards. On 98 of them, it says “intervention group” and on 98 “control group.” A unique ID is assigned to each card. The participants draw the cards themselves. This gives them the feeling that they have made their own decision and makes it easier for them to accept it, even if they do not end up in the desired group.

### Implementation {16c}

The drawing of the participants takes place during the initial appointment for the informed consent. Since the participants come continuously and from all over Lower Austria, interviews are conducted both in person and via video calls. In order to make the ballot box drawing online and offline comparable, a computer-based drawing will take place in both cases, i.e., similar to a memory game. The test persons turn over one (of 196) cards and thus find out in which group they participate and, at the same time, receive their ID for the study. The drawing takes place without putting the cards back.

The maximum group size is 14 participants. When an intervention or control group is full, the recruitment of the next group will be started. The ID numbers are therefore not consecutive within the groups, but random. There is no upper limit regarding the number of participants for the first recruitment date.

## Assignment of interventions: blinding

### Who will be blinded {17a}

Blinding of trial participants will not be possible because of the obvious differences between the intervention group and the control group. Blinding will be implemented for the medical staff and the outcome assessors of the measurements and the following analyses (see {19}).

### Procedure for unblinding if needed {17b}

It is an open-label study and therefore unblinding will not occur.

## Data collection and management

### Plans for assessment and collection of outcomes {18a}

During the online registration, contact data of all interested individuals will be collected. The data will be deleted if the person disagrees to participate in the trial.

All parameters as described in the subsection “Outcomes (see {12})” will be taken in a group testing setting with appropriate Coronavirus SARS-CoV-2 (COVID-19) safety measures, e.g., testing, vaccination, hygiene procedures by the staff of the St. Pölten University of Applied Sciences either at the university itself or at the Landesklinikum Wr. Neustadt, Hollabrunn, or Mauer/Amstetten.

The measurements will take place in the morning in a fasting state and with anthropometric measurements with light clothing. Anthropometric measurements will be conducted by dietitians. Blood pressure and blood samples will be taken by nurses. Fresh blood samples will be taken by the staff of the St. Pölten University of Applied Sciences, centrifuged within a timeframe of twenty minutes, and analyzed within a timeframe of 3 h maximum after the centrifugation by the University Hospital St. Pölten. The laboratory of the University Hospital St. Pölten is ISO 9001-certified and will handle all samples according to its quality management.

### Plans to promote participant retention and complete follow-up {18b}

To minimize loss during the follow-up, and in order to promote participation in the follow-up measurements, the same strategy is implemented as for compliance with the intervention measurements (see {11c}): as convenient as possible, reminders 48 h ahead of the measurement, possibility to discuss the measurements’ results with the dietitian, and a financial compensation paid after the last measurement.

During the follow-up, participants of the intervention group can use the instant messaging service and continue their communication with the group members and the moderator, hence, without support of the study team. The moderator will be instructed to motivate the group members to participate in the follow-up measurement. The relationship of trust and social support in the groups as well as with the study staff will support a higher degree of complete follow-up.

### Data management {19}

The participants agree that for the purpose and in the course of this study, the St. Pölten University of Applied Sciences will collect and process the following personal data: name, age, gender, education, income, occupational situation, housing situation, migration background, personality characteristics, clinical information such as age at diabetes diagnosis, social support, self-efficacy, depression, quality of life, diabetes knowledge, treatment adherence, diet and exercise behavior, nicotine and alcohol consumption, diabetes self-management, medical consultations during the last 6 months, HbA_1c_, fasting glucose, total cholesterol, HDL, LDL, triglycerides, blood pressure, body weight and height, waist circumference, and body composition.

Furthermore, the participants agree that the University Hospital St. Pölten may transmit the following data in indirectly personalized (pseudonymized or encrypted) form to the St. Pölten University of Applied Sciences for the purpose of analyzing whether the intervention is effective with regard to the defined parameters compared to conventional therapy: HbA_1c_, fasting glucose, total cholesterol, HDL, LDL, triglycerides.

The participants’ data will be pseudonymized and assigned a fixed identification code. This code will be stored safely in a password-protected file, accessible only by the study coordinator (EH) and central staff members (UH, AEZ). The key for the coding is kept separately and inaccessibly by the study coordinator. Thus, it is only possible for persons who are entrusted with the evaluation of the data to infer the identity of the participants.

Data acquisition and data processing are performed using commercial software. All data obtained are stored in computer files in encrypted form. The coding is done by means of consecutive numbering, from which only the allocation to the corresponding group, but in no case a conclusion as to the identity of the participant is possible.

Data will be stored and analyzed via the statistical software package SPSS at servers of the St. Pölten University of Applied Sciences, only used for this special study and not be passed on to third parties. The handling of the data complies with the European General Data Protection Regulation, the Austrian Data Protection Act, and the recommendations of the Ethics Committee of Lower Austria.

To be able to merge the data from the paper-and-pencil questionnaires, anthropometric measurements, and blood pressure data, a defined coding system will be used. Therefore, the study coordinator will take part in every measurement and organize the participants and coding system. Hence, every participant will get his or her identification code and the questionnaires will be marked with this code at the beginning of each measurement. The identification code ensures that the study staff do not know to which group each participant belongs. Paper-and-pencil questionnaires will be checked by the study staff in terms of completeness and correctness related to the specifications of the questionnaires. These data will be transferred into SPSS manually and controlled by another member of the study team. Results of the biochemical analysis will be mailed via encrypted Excel files from the University Hospital St. Pölten to the St. Pölten University of Applied Sciences and imported into SPSS by the study coordinator. Blood samples will be disposed of and Excel files will be deleted after integration into SPSS. Furthermore, the St. Pölten University of Applied Sciences performs data back-ups on a daily basis. All signed informed consent forms as well as all collected paper-and-pencil sheets will be stored in a locked place, accessible only to the study coordinator and a central staff member (UH). All data will be stored at the St. Pölten University of Applied Sciences for ten years after the study’s end. After that, all data will be destroyed.

To ensure data quality, the study coordinator will control the data regularly including checking the range of values or double data entry.

Data protection concerning the instant messaging tool will be met by using the instant messaging communication tool *mattermost* (https://mattermost.com/). *mattermost* has been chosen for its clear terms and conditions and data handling strategy. The open-source software *mattermost* was chosen because it can be operated securely and privately, as we will run it on-premise and not as a cloud solution. *mattermost* will be hosted as a software solution in an ISO 27001:2013 certified data center in Vienna for the duration of the project. *mattermost* provides a holistic messaging solution that allows to communicate securely and efficiently via 1:1 and group messaging, meeting, and file sharing as well as audio/video integrations. The data export based on Docker is conducted in the Viennese data center and made available only to selected members of the study team (EH, AEZ, JG). The data are cleaned, and unstructured communication data will be structured using content analysis and MAXQDA (see {12}). Subsequently, these data will be pseudonymized and transferred into SPSS for further analysis.

Results of all analyses will be reported in an aggregated and strictly anonymous form.

### Confidentiality {27}

All collected data will be kept confidential as described in “Data management” (see {19}). Only the study coordinator and central staff members (UH, AEZ) can open the password-protected file to link personal information and code. The final pseudonymized dataset in SPSS will be used by the study staff for analyses, publications, and reports only for this study.

### Plans for collection, laboratory evaluation, and storage of biological specimens for genetic or molecular analysis in this trial/future use {33}

Not applicable as no biological specimens for genetic or molecular analysis will be collected.

## Statistical methods

### Statistical methods for primary and secondary outcomes {20a}

Data will be analyzed using IBM SPSS Statistics 26 or greater (IMB Corporation, Armonk, NY, USA). The significance level will be set at 5%. Generally, metric data will be checked for normal distribution using a histogram of each variable, as well as for skewness and kurtosis. To illustrate descriptive parameters, the arithmetic mean and the 95% confidence interval will be stated for metric data. Interval variables or nominal scaled variables will be shown as frequencies. Repeated measures ANCOVA will be used to compare the control and intervention groups concerning primary and secondary outcome parameters. Non-normal metric data will be analyzed using the Mann-Whitney *U* test, the Kruskal-Wallis signed rank test, or the Friedman-Test [73].

### Interim analyses {21b}

There will be no planned interim analysis or stopping guidelines for medical reasons besides the exclusion criteria (see {10}) because no potentially harmful outcomes are expected based on the peer-supported IMS intervention. Nevertheless, participants can be excluded from the intervention group if they do not follow the rules of interaction set and agreed upon in the IMS group (see {11b}).

### Methods for additional analyses (e.g., subgroup analyses) {20b}

As we want to assess the association between specific personality traits and the benefits of peer-supported IMS, we use validated and published questionnaires, which we repeatedly distribute to all experimental and control groups at different times of the study.

In addition to the observed communication behavior, we use the information from the questionnaires (see {12}) to test the hypotheses and in particular related to (H2) diabetes self-management behaviors, (H3) quality of life of participants, and (H4) medication adherence as well as (H5) the level of extraversion.

### Methods in analysis to handle protocol non-adherence and any statistical methods to handle missing data {20c}

All participants’ data will be analyzed following the intention-to-treat principle. Scores for scales with at least 80% of items responded will be analyzed. For these cases, the nearest neighbor method will be used to replace missing data points. Furthermore, we will check the randomness of missing data by calculating the MCAR (missing completely at random) classification.

### Plans to give access to the full protocol, participant-level data and statistical code {31c}

It is not planned to give third parties access, neither to the full protocol, nor the participant data, nor the statistical code.

## Oversight and monitoring

### Composition of the coordinating centre and trial steering committee {5d}

The complexity of diabetes therapy demands interdisciplinary teams and innovative treatment approaches. This consortium meets these requirements and combines life sciences, nutritional sciences and dietetics, human medicine, psychology, and with media economics, communications, and sociology in a unique constellation. Responsibilities are assigned on the basis of the expertise of the respective study team members. Responsible persons for the following areas are defined as relevant to the study: Instant Messaging Service Interventions, Dietetics, Medical Measurements, Recruitment, and Biochemical Analyses and Statistics. In addition, two persons are available as supervisors for the areas of Nutrition and Communication, respectively, and a medical doctor is also part of the study team.

Meetings of the entire study team are held every 2 months or more frequently when meetings are needed or in subgroups. In addition, experts are available on request. Decisions are made democratically. The study coordinator has the final responsibility.

Ethics approval has been obtained from the local Ethics Committee prior to the start of the study (see {24}). Technical know-how is mainly outsourced to the service units of the instant messaging service *mattermost*.

### Composition of the data monitoring committee, its role, and reporting structure {21a}

A Data Monitoring Committee (DMC) has been established (EH, AEZ, UH, JG).

On the one hand, the role of the committee members (AEZ, JG) is to conduct weekly checks of the communication protocols and to identify potential errors in measurement or possible anomalies in the behaviors of participants and moderators.

In addition, members of the DMC (EH, UH) routinely check that adequate procedures are in place to ensure that the data remain confidential and that there are no data breaches. If security problems are identified, the Data Monitoring Committee informs the other study members or, depending on the severity of the problem, also the funding agency, and takes action to fix the problem.

### Adverse event reporting and harms {22}

Although we consider specific risks for participation very low, a system for collecting, assessing, reporting, and managing adverse events will be implemented. Every potential adverse event will be documented in detail. The study coordinator will immediately inform the Ethics Committee of Lower Austria and the study’s insurance company if necessary.

If a problematic situation arises in an IMS group such as bullying, depression, racial/sexist/homophobic, etc. statements, extensive sharing of fake news, commercials, and the like, the moderator can try to solve it using the communication strategies learned during the training, discuss it with the dietitian and/or other moderators, or hand over the problem to the dietitian and/or the study staff. Furthermore, the study team will control the communication data for problematic situations at regular intervals. The dietitians will address this topic during the monthly meetings with the moderators as part of the process evaluation.

### Frequency and plans for auditing trial conduct {23}

The whole consortium meets regularly and is dependent on the project’s tasks. The meetings will be conducted at least twice a year and a maximum of every 2 months.

The study coordinator (EH) and central staff members (UH, AEZ) meet twice a month to review the trial conduct.

The Data Monitoring Committee (DMC) meets as described in the subsection in “Composition of the data monitoring committee, its role, and reporting structure” (see {21a}).

The study coordinator reports to the Ethics Committee as well as to the funding organization at least once a year. If adoptions of the study protocol will become essential, further notifications will be made to these organizations by the study coordinator.

### Plans for communicating important protocol amendments to relevant parties (e.g., trial participants, ethical committees) {25}

Approval for protocol modification will be sought for from the Ethics Committee of Lower Austria as well as the funding organization (Gesellschaft für Forschungsförderung Niederösterreich m.b.H, Austria) using the report form of the Ethics Committee and the funding organization. Upon approval of all changes, we will update the clinical trial registry. If protocol modifications differ from what was explained to participants when signing the informed consent, the participants will be informed immediately about all changes and re-consent will be sought by a study staff member (dietitian or nutritionist).

### Dissemination plans {31a}

The consortium has developed a dissemination plan which defines all relevant stakeholders and appropriate dissemination strategies as well as authorship guidelines [74] visualized in a GANTT chart including when to conduct which dissemination activity, and another tool for the documentation of all activities. The dissemination plan includes scientific as well as general dissemination activities.

All dissemination activities will be documented and reported by the responsible consortium member. When it comes to scientific dissemination, the study protocol and the study results will be published in peer-reviewed open access journals. The study and its results will be presented at scientific conferences. Various activities are planned to disseminate generally, e.g., at special events like the “European Researchers’ Night” or at the “World Diabetes Day”, study website posts, press releases, or radio and TV contributions.

## Discussion

Type 2 diabetes mellitus is one of the major global causes of disability and mortality. During the past few decades, the prevalence of diabetes increased dramatically all over the world and rising numbers are expected for the next decades [1, 2].

Additionally, data indicate that the COVID-19 pandemic will worsen the global diabetes burden because of the bi-directional relationship between these two diseases: on the one hand, diabetes is associated with a poor prognosis of COVID-19, and COVID-19 patients with diabetes are more likely to face uncontrolled hyperglycemia or acute hyperglycemic crisis. On the other hand, a recent meta-analysis with approximately 3700 participants shows a pooled proportion of 14.4% for newly diagnosed diabetes in hospitalized COVID-19 patients [75]. Furthermore, the COVID-19 pandemic is suspected to have long-term effects on the prevalence of type 2 diabetes mellitus because of the lockdowns in almost every country. This unique situation has led to harmful effects such as increased calorie intake, reduced physical activity, increased screen time, weight gain, depression, boredom, disrupted sleep pattern, anxiety, and stress [76, 77]. The majority of these effects are well-known risk factors for the development of type 2 diabetes mellitus [10].

This situation highlights the importance of appropriate diabetes management. DSMES is a key component of successful diabetes management and it is highly relevant to maintain the positive results in the long run. Therefore, DiabPeerS aims to improve diabetes self-management leading to better glycemic control by implementing a peer-supported IMS intervention in addition to standard care. IMS provides several benefits:

- IMS can be used time- and location-independently, which leads to higher participation rates of people with type 2 diabetes mellitus in diabetes self-management support offers.
- IMS has a low threshold when exchange with others is needed.
- IMS support and exchange can be provided immediately.
- the majority of patients with diabetes already use IMS, which means that no technology barrier is perceived.
- IMS groups can be supervised by different health professionals at the same time or used to share workload or offer additional support. The additional support is an option of information verification, which increases the quality of the peer support.
- IMS can help to lower costs for participants as well as for the healthcare/social system.

To ensure positive effects of peer-supported IMS interventions, participants, moderators, and health professionals should undergo adequate training because IMS demands very specific communication, e.g., engaging and motivating wording, emojis, or short messages. Therefore, the DiabPeerS study has developed specific trainings for participants, moderators, and dietitians. Information concerning these trainings will be published after the end of the study.

Based on the multifactorial origin of type 2 diabetes mellitus and the need for holistic therapeutic approaches, the DiabPeerS study consists of an interdisciplinary team and looks at the disease requirements as well as the peer-supported IMS intervention from different scientific angles by adopting a mixed-methods approach. On the one hand, the study team will gain new insights concerning effective strategies to support diabetes disease management and improve disease-related outcomes. On the other hand, the IMS strategy and the trial outcomes can be analyzed in detail, e.g., topics can be identified which lead to increased discussion and exchange of participants or in-depth analysis of the results of the quantitative data.

Besides the undoubted negative consequences of the COVID-19 pandemic, people all over the world are now used to communicating online, which has led to a digital boost in the healthcare sector. As a consequence, new digital therapeutic and/or supportive approaches to various diseases - especially non-communicable diseases - will play an even greater role in the future. DiabPeerS is intended to make a relevant contribution to future high-quality online services for patients.

## Trial status

This is the protocol version 6 from 21.11.2021. Recruitment has started in August 2021 and will end in September 2022.

## Abbreviations

ADA: Aktive Diabetiker Austria
ANCOVA: Analysis of covariance
BFI-2: Big Five Inventory German
BIA: Bioelectrical Impedance Analysis
COVID-19: Coronavirus SARS-CoV-2
DiabPeerS: Improving glycemic control in patients with type 2 diabetes mellitus through a peer support instant messaging service intervention
DDS: Diabetes Distress Scale
DKT: Diabetes Knowledge Test
DMC: Data Monitoring Committee
DSM-IV: Diagnostic and Statistical Manual of Mental Disorders
DSME: Diabetes self-management education
DSMES: Diabetes self-management education and support
EUR: Euro
FFQ: Food frequency questionnaire
FoKo database: Folgekosten database
F-SozU: Fragebogen zur Sozialen Unterstützung
GSE: General Self-Efficacy Scale
HbA_1c_: Glycated hemoglobin
HDL: High-density lipoprotein
IMS: Instant messaging service
IPAQ-SF: International Physical Activity Questionnaire Short Form
IT: Information technology
LDL: Low-density lipoprotein
MCAR: Missing completely at random
MET: Metabolic equivalent of task
mHealth: Mobile Health
ÖDV: Österreichische Diabetikervereinigung
PHQ-9: Patient Health Questionnaire-9
RCT: Randomized controlled trials
SDSCA-G: Summary of Diabetes Self-Care Activities German
SF-12: Short-Form-Health Survey
TV: Television
USD: US dollar
